# Cognitive Bias in Adult Zebrafish (*Danio rerio*): A Systematic Review

**DOI:** 10.3390/vetsci12010071

**Published:** 2025-01-20

**Authors:** Valentina Gazzano, Asahi Ogi, Francesca Cecchi, Maria Claudia Curadi, Maria Marchese, Angelo Gazzano

**Affiliations:** 1 Department of Veterinary Sciences, University of Pisa, 56124 Pisa, Italy; valentina.gazzano@unipi.it (V.G.); francesca.cecchi@unipi.it (F.C.); maria.claudia.curadi@unipi.it (M.C.C.); angelo.gazzano@unipi.it (A.G.); 2 Neurobiology and Molecular Medicine, IRCCS Stella Maris Foundation, 56128 Pisa, Italy; maria.marchese@fsm.unipi.it

**Keywords:** zebrafish, cognitive bias, animal welfare, personality

## Abstract

Objective: In recent years, the use of zebrafish (*Danio rerio*) as laboratory models has significantly increased. Ensuring their welfare is crucial, with the cognitive bias test emerging as a valuable tool to assess their emotional state. This systematic review examines the application of the cognitive bias test in zebrafish research. Data: The review adhered to PRISMA guidelines. Sources: A systematic search was conducted in scientific databases, including PubMed and Scopus, yielding 30 articles. Additionally, one abstract identified through bibliographic screening of selected papers was included. Study Selection: Six studies were selected for analysis, and their quality was assessed using SYRCLE’s risk-of-bias tool. Results: Three experimental models were employed to conduct the cognitive bias test in zebrafish, focusing on the relationship between their affective state and environmental conditions, cognitive abilities, and life-history strategies but none of the studies adequately reported the techniques employed to reduce bias. Discussion: The cognitive bias test proved effective in assessing the emotional state of zebrafish; however, methodological biases may have influenced the results. Conclusion: The cognitive bias test offers valuable insights into how emotional states affect cognitive abilities, responses to environmental changes (e.g., physical enrichment), and stress responses. Further research addressing methodological limitations is necessary to validate these findings and improve data reproducibility.

## Simple Summary

Recent years have seen a progressive replacement of mammals as laboratory animals with other species, such as zebrafish (*Danio rerio*). Safeguarding the welfare of these animals remains imperative, according to the latest scientific findings. Evaluating an animal’s welfare must include consideration of its emotional state, which can be assessed using the cognitive bias test. This manuscript examines existing research on the application of cognitive bias tests to zebrafish, highlighting its potential as a tool for ensuring their well-being. A systematic review conducted using the PubMed and Scopus databases identified 6 scientific papers addressing the topic. All the research conducted so far confirms, despite presenting methodological biases, the possibility of using the cognitive bias test in zebrafish to assess their emotional state, reinforcing its value in promoting welfare for this species.

## 1. Introduction

Recent years have seen a progressive replacement of mammals as laboratory animals with other species like the zebrafish (*Danio rerio*) which is gaining popularity in a wide range of studies, including toxicology, pharmacology, neuroscience, and behavioral medicine [1,2,3]. The high similarity to human genes, easy and low-cost maintenance, high reproductive rate, and the transparency of embryos and larvae make this animal an ideal model [3]. Moreover, the EEC Directive 2010/63/EU emphasizes the importance of animal welfare, aiming to “raise the minimum standards for their protection in line with the latest scientific developments”. However, evaluating animal welfare is a complex process that requires a comprehensive understanding of various factors, from assessing an animal’s health and meeting its essential physiological needs to considering its interaction with its physical [4] and social environment.

The recognition of sentience (the ability to feel and experience emotions such as joy, pleasure, pain, and fear) [5] in many animal species has further highlighted the need to consider the emotional state of animals to ensure genuine welfare. This, however, raises the challenge of scientifically assessing emotional state. According to the American Psychological Association (APA), emotion is defined as “a complex reaction pattern, involving experiential, behavioral and physiological elements.” (APA Dictionary of Psychology). Classical theories suggest that emotional experiences comprise three components: subjective experience, physiological, and behavioral reactions, but interpreting these components in non-human animals remains challenging [6]. It has long been recognized in human psychology that cognitive processes both influence and are influenced by emotional states. Researchers have sought parallels between humans and animals in their studies of emotion and cognition [7,8,9,10], which could allow for the identification of objective measures of cognitive performance as indicators of emotional state. For example, studies on humans have shown that emotional states influence cognitive processes such as attention, memory, and judgment [9].

Most animal studies on cognitive biases have focused on “judgment biases” [11], as defined by Mendl et al. [6]; this refers “to the propensity of a subject to show behavior indicating anticipation of either relatively positive or relatively negative outcomes in response to affectively ambiguous stimuli. Such propensities can be operationally defined as ‘optimism’ or ‘pessimism’, respectively”.

Although cognitive bias has been observed across various species [12], little is known about this topic in fish [13]. Research has revealed that zebrafish exhibit consistent and individual differences in behavior, which are often referred to as “personality traits” like boldness and shyness [14] but also aggressiveness [15], activity level [16], and sociability [17]. Personality and cognitive bias are closely linked [18]. Indeed, the interplay between personality and cognitive bias underscores the complexity of behavior and how intrinsic traits shape perceptions and judgments in different situations. For this reason, “cognitive bias” and “judgment bias” are often terms used interchangeably [19]. However, the literal definition of judgment bias is not the same as cognitive bias because cognitive biases can lead to judgment biases, but cognitive biases do not encompass just judgment. For an in-depth digression on possible definitions of cognitive bias, see Trimmer et al. [20].

In non-human species, the term “cognitive bias”—and we would also say “judgment bias”—has been broadly applied to refer to decisions influenced by emotions [20]. It follows that the affective state or, by extension, the “personality” of zebrafish could impact the outcome of the cognitive bias test. Both personality traits and emotional states are known to influence zebrafish [21]. It is well-known, in fact, that fear induces shoal cohesion in zebrafish [22]. Moreover, personality traits like boldness and shyness are also linked with the ability of the organism to cope with stress [23]. Indeed, according to Koolhaas et al. [24], there are two major types of coping styles, proactive (bold) and reactive (shy), and the existence of these styles in fish is now widely acknowledged [25]. Since behavioral responses can be opposite depending on the coping style—freeze (reactive) versus fight/flight (proactive)—personality traits should be taken into consideration every time we assess animal welfare through behavioral tests [23].

Baker et al. [26] investigated whether stress response and cognitive abilities differ among personality types. In certain contexts, such as stress-coping strategies, personality types are particularly pronounced [27]. It is, above all, the type of behavior chosen rather than its intensity that differentiates one individual from another [28]. Based on this evidence, researchers studied zebrafish with two distinct personality types: those with low-stationary behavior (LSB) (proactive style) and those with high-stationary behavior (HSB) (reactive style), dividing them into two groups. The results revealed that both groups exhibited high levels of freezing behavior, but reactive individuals displayed this behavior more rapidly and for a longer duration. In recall tests conducted 96 h later, reactive individuals (HSB) exhibited a greater amount of freezing time compared to proactive individuals (LSB), indicating stronger memory retention [26]. These findings suggest that cognitive bias provides valuable insight into emotional states and overall welfare.

The cognitive bias test, first applied in rats by Harding et al. [29], has been used to evaluate emotional states in various animal species, including dogs [11], cats [30], mice [31], chickens [32], and pigs [33]. Judgment bias trials involve two main tasks: active choice (go/go), where animals select between two responses, and go/no-go, where they choose to perform or suppress a response. Animal responses are quantified as proportions (e.g., percentage of specific responses) or latencies (e.g., time taken to respond). Factors influencing these tests are reviewed by Lagisz et al. [34].

The aim of the present manuscript is to identify, through a systematic review of available studies, the different cognitive bias paradigms and their possible use in zebrafish.

## 2. Materials and Methods

**Figure 1:**
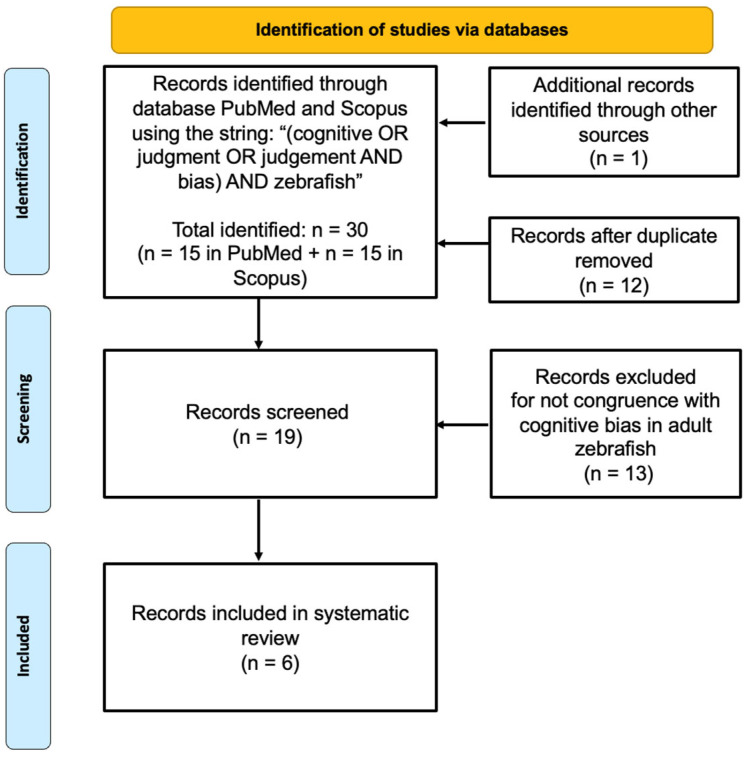
PRISMA flow diagram of the literature search process.

This review was conducted following the Preferred Reporting Items for Systematic Reviews and Meta-Analysis (PRISMA) guidelines [35] (Figure 1).

The systematic review protocol was not registered. The research question was formulated using the P.I.C.O.T. model (Participant, Intervention, Comparison, Outcome, Time period) [36]. Specifically, the outcome (O) sought was to identify various cognitive bias paradigms (I) and their potential applications (C) in adult zebrafish (P) up until 21 March 2024 (T). A systematic search of the electronic bibliographic databases PubMed and Scopus was conducted.

One of the authors (V.G.) received specialized training in bibliographic research methodologies. The search strategy utilized the following query: (cognitive OR judgment OR judgment AND bias) AND zebrafish.

Exclusion criteria included abstracts, theses, and dissertations. Additionally, reference lists of the selected studies were manually examined to identify further relevant studies.

The screening process was carried out independently by two reviewers, V.G. and A.O., who evaluated titles and abstracts. If both reviewers agreed that a study did not meet the inclusion criteria, it was excluded. Any discrepancies were resolved by consulting a third reviewer, A.G. The full texts of papers that met the initial screening criteria were subsequently assessed by the same reviewers, with any conflicts resolved through consensus with the third reviewer. The three reviewers are qualified veterinary behaviorists.

For synthesizing findings, a narrative approach was employed, focusing on key factors such as age, genotype, pharmacological treatment, and cognitive bias test methodology.

All selected studies underwent a risk-of-bias assessment using the Systematic Review Center for Laboratory Animal Experimentation’s (SYRCLE’s) risk-of-bias tool [37]. This assessment was independently conducted by V.G. and A.O. and disagreements were resolved through consensus-oriented discussion.

## 3. Results

The systematic review identified thirty articles. After removing twelve duplicates and including one abstract from the XV European Congress of Ichthyology, identified through bibliographic screening, nineteen articles were eligible for full-text screening. Of these, twelve were excluded for not meeting the study’s objectives, and one because of lacking a test for cognitive bias, leaving six studies included in the systematic review.

A total of six articles were therefore included, of which one study demonstrated the paradigm of the cognitive bias test [38], two studies correlated the affective state with life-history strategies [39,40], two studies evaluated the relationship between environmental factors and the affective state [41,42], and one study focused on the effects of emotional state on cognitive abilities [43].

The inter-rater reliability of data screening, measured using Cohen’s kappa coefficient (k = 0.883), indicated almost perfect agreement.

The six papers found to be consistent with the aim of this review after the selection process are reported in Table 1.

### How to Test Cognitive Bias

Different behavioral tests are used to assess the judgment bias in relation to the affective state or personality in zebrafish.

Generally, they are structured in three phases: habituation phase, training phase, and test phase.

In the habituation phase, individual fish were allowed to explore the entire apparatus. In the training phase, a specific area, marked by a particular color or context, is associated with positive reinforcement, punishment, absence of reinforcement, or variation in the quality of reinforcement. Finally, in the test phase, the fish’s decision-making process is evaluated.

**Figure 2:**
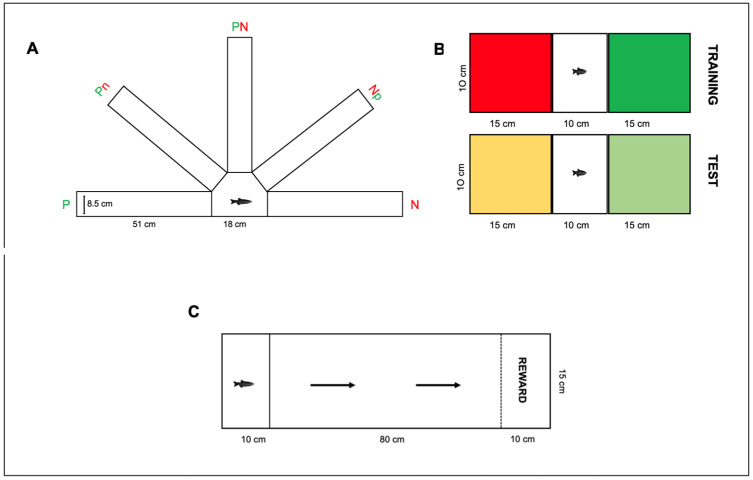
Test paradigms. (**A**): Paradigm 1, Espigares et al.,2021 [40]; Espigares et al., 2022a [38]; Espigares et al., 2022b [39]; Wojtas et al., 2015 [41]; (**B**): Paradigm 2, Buenhombre et al., 2023 [42]; (**C**): Paradigm 3. Tan et al., 2020 [43].

The most used model (paradigm 1; Figure 2A), adopted in four papers [38,39,40,41], was a five-arms device, with each arm ending with a colored card. The arms positioned at 180° were used for the training phase [Positive (P) and Negative (N)], presented a full-colored card (green or red), and were associated with reinforcement or punishment. The other three arms, considered ambiguous, were used for the test phases: they were positioned near the P arm (NP), in the middle between the P and N arm (A), and near the N arm (NN), presenting mixed-colored cards with different color proportions based on the closest reference. In this paradigm (a go/no-go task), the latency to enter each ambiguous arm was evaluated according to the hypothesis that an optimistic individual is one who takes less time to enter the arm in an ambiguous position near the negative one. From this supposition, a judgment bias score (JBS) can, therefore, be assigned for each fish based on whether they were classified as either pessimistic (JBS>50) or optimistic (JBS < 50) [38].

The second paradigm (Figure 2B) was used by Buenhombre and colleagues [42]. In this go/go model, a modification of the conditioned place preference test [44], the device adopted was a tank consisting of two equal areas marked with different colors, divided by a central section. During the training phase, only one area was associated with positive reinforcement, while no reinforcement was associated with the other area. For the test phase, the same device was used but with ambiguous colors to mark the areas, and the time spent in the ambiguously colored area was evaluated, assuming that an optimistic individual would be one who spends the most time in the area marked with the ambiguous yellowish color similar to the reference with positive reinforcement.

Tan and colleagues [43] used a device (Figure 2C) formed by a channel where the fish must travel through to reach a reward administered at the end of it.

In this paradigm (a go/no-go task), during the training phase, the fish had to swim along the channel to reach a specific reward (flakes or shrimp). In the test phase, the speed at which the fish swam to reach the reward was evaluated after an unexpected reduction (from shrimp to flakes) or increase (from flakes to shrimp) in reward value. The hypothesis was that an optimistic individual would show a smaller difference in the time taken to reach the reward zone when the reward value decreases and a larger difference in the time taken when the reward value increases.

## 4. Purpose of Using Cognitive Bias

### 4.1. Correlate Between the Affective State and the Life-History Strategies

Testing cognitive bias was used by Espigares and colleagues in their two studies [39,40] to correlate the affective state with the life-history strategies (LHT), a framework that explains how individuals allocate energies between different strategies to enhance their evolutionary success throughout their lifespan. These strategies are typically divided between somatic effort, which involves investing in oneself for future benefit, and reproductive effort, which focuses on immediate reproduction with an emphasis on the quality of offspring [45]. Two types of life-history strategies have been identified: fast and slow [45,46,47]. In unfavorable and unexpected environments, individuals implement a fast LHT [39,45], exhibiting premature maturation, small body size, and lower life expectancy [47]. Additionally, they allocate more energy to current reproduction and tend to have a reduced ability to cope with stressful situations [45,46]. On the other hand, in an unharmed and expected environment, individuals execute slow LHT [39,45] with late maturation, larger body sizes, and longer life expectancy [47]. They focus on future reproduction, aiming for higher offspring quality with greater chances of survival [46].

In the first study, Espigares and colleagues [39] investigated whether an individual’s affective state (optimist or pessimist) influenced reproductive strategies under different environments. They assess 72 four-month-old female zebrafish using a judgment bias assay to categorize them as optimistic or pessimistic.

Afterward, the fish were exposed to unpredictable chronic stress for 17 days before being sacrificed, and their ovarian structures were analyzed. The ovarian structure was divided into four stages based on follicular maturation: the primary growth stage identified by primary oocytes; the cortical alveolus stage, where the formation of the vitellin envelope and yolk deposition begins; the vitellogenic stage, characterized by an increase in size due to yolk accumulation; and the mature stage, with nuclear dissolution and yolk bodies [48]. The authors found that chronic stress can cause alterations in reproductive outcomes, as evidenced by the fact that females exposed to chronic stress showed ovaries with a high number of immature follicles. However, pessimistic females had ovaries with a higher vitellogenic area compared to optimistic females, providing clear evidence of differential reproductive investment based on affective state.

In the second study, Espigares et al. [40] evaluated the potential link between short telomeres, affective state, and life-history theory. Telomeres are terminal regions of chromosomes that protect them from damage or fusions with other chromosomes. The shortening of these telomeres is prevented by telomerase, an enzyme found in somatic cells. However, during life, a physiological shortening of this region occurs even in the presence of telomerase. In humans, this shortening can be accelerated by various events such as chronic physiological stress and emotional-related disorders. Based on evidence from human medicine, this study used a *tert* homozygous zebrafish strain with shorter-than-expected telomeres and, consequently, a short life expectancy. For this study, 10–12 fish of both WT and *tert*−/− aged 4 and 9 months were tested in a behavioral assay to evaluate their latency to enter each arm of the testing apparatus. The results showed that zebrafish *tert*−/− mutants display more pessimistic-like behavior compared to WT zebrafish of the same age.

### 4.2. Evaluation of the Relation Between the Environmental and the Affective State

Environmental factors and housing conditions also significantly influence zebrafish affective states and decision-making processes. Particular attention has been given to whether environmental enrichment can also influence the animal’s affective state [42] and so, the possibility of using the cognitive bias test to assess welfare conditions in housed animals [41]

Wojtas et al. [41] tested two different groups of fish: one group was housed in an enriched tank containing hiding places and exposed to soft light, and the other group was housed in a barren tank exposed to strong light. At the end of the test, the zebrafish of the first group exhibited more exploratory behavior, spending more time in the A and N arms. The second group spent more time in the P arms and avoided the N ones (Figure 2A). The authors concluded this type of test should be taken into consideration as a tool in further fish welfare studies, especially useful when the influence of living conditions cannot be examined in a direct way.

Buenhombre et al. [42] focused their studies not only on the effect of environmental enrichment but also on the way it is provided, on the zebrafish’s affective state. Thus, six groups of fish were tested after experiencing housing manipulation: two groups underwent a sudden gain or loss of enrichment, respectively., two other groups experienced a gradual gain or loss of enrichment, and finally, the last two groups were maintained with the same environmental or barren enrichment, whichever it was. Fishes that experienced a sudden gain in environmental enrichment exhibited more pessimistic behavior, spending less time in the area marked with the ambiguous yellowish color (like the reference with positive reinforcement), compared to those kept in consistently barren or enriched conditions.

This is likely due to the increased unpredictability of stimuli, which may induce a negative affective state, as demonstrated in numerous studies [49,50,51]. In fact, based on the appraisal theory, which identifies emotion as a cognitive process of evaluation, a set of appraisal components link the individual’s affective state to the surrounding environment [49,52,53]. The predictability, and thus the controllability, of an event is one of the appraisal components described in animals and plays a very important role in the emotional-like state [49,52].

### 4.3. Effects of Emotional State on Cognitive Abilities

Individual variations in affective states, such as optimism and pessimism, influence cognitive processes like perception, learning, memory, and decision-making [27].

For example, incentive contrast studies reveal how unexpected shifts in reward quality or quantity affect an animal’s behavior [43]. In fact, in the context of incentive contrast, two different phenomena can take place [43,54]: the first, termed successive negative contrast (SNC), occurs when there is an unexpected reduction in quality or quantity reward compared to what the subject was accustomed to [43,54,55,56]. In this case, individuals reduce their instrumental or consummatory behavior and exhibit frustration or depression-like behavior [43,54,55,56]. On the contrary, when there is an increase in reward, successive positive contrast (SPC) occurs [43,54].

It has been demonstrated in various animal species that affective state can influence sensitivity to reward shifts [43,54,55], making it reasonable to assume that this could be used as a tool for assessing animal welfare, particularly in laboratory animals. Based on this, Tan and colleagues [43] hypothesized that fish in a positive affective state would show less SNC and more SPC. Initially, a housing preference test was conducted to assess the affective state of the subjects, with the assumption that fish housed in a preferred environment were in a positive affective state. Then, four groups were tested based on the housing environment and the reward received: enriched/flakes, enriched/shrimp, barren/flakes, and barren/shrimp. Contrary to expectations, neither group, those experiencing a reduction or increase in reward value, showed a significant difference in the speed with which they swam the canal, with no evidence of SNC or SPC. Furthermore, there was no difference between the groups housed in the enriched tank and those housed in the barren tank, suggesting that the choice of enrichment, assumed to promote a positive state, did not influence sensitivity to reward.

### 4.4. Reporting Quality and Risk of Bias Assessment

The risk of bias assessments for the six reviewed articles are summarized in Table 2. A “yes” judgment (Y) indicates a low risk of bias, while a “no” judgment (N) signifies a high risk of bias. If the report lacks sufficient detail to assess the risk appropriately, the judgment is marked as “unclear” (NC).

None of the studies adequately reported the techniques employed to reduce bias. In all cases, the method used to randomize animals into treatment groups (A) was not clearly described, nor was it specified whether animals were randomly placed in the facility (D). A clear indication of the similarity between animal groups at the start of the experiment was provided in 50% of the studies (B). Bias was most effectively minimized by including all animals in the analysis (H) in 66.6% of the studies. Furthermore, 83.3% of studies reported both the primary and secondary outcomes of the research and made the study protocol available (I). Half of the studies (50.0%) did not report any other significant issues that could introduce bias (J). Regarding whether caregivers and/or investigators were blinded to the interventions received by each animal during the experiment (E), the nature of the experiments inherently made it difficult to eliminate this bias. However, the potential influence of this limitation could be considered minimal.

## 5. Discussion

The adoption of non-mammalian species in research necessitates rigorous welfare evaluations. The cognitive bias test offers researchers a unique opportunity to “step into the shoes” of the animal, providing insight into how individuals perceive their relationships with the social and physical environment. This approach yields significantly more comprehensive information compared to the traditional evaluation of hormonal and zootechnical parameters, which have been predominantly used in various species to assess welfare levels. Although the test is limited by the fact that animals make choices based on present circumstances without the ability to predict long-term outcomes, it remains a valuable tool for safeguarding animal welfare.

This review highlights the application of the cognitive bias paradigm in zebrafish, demonstrating its utility in assessing welfare conditions in this species. Adapting this technique to an aquatic species required the development of specialized devices capable of creating ambiguous stimuli, thereby allowing zebrafish to choose whether to engage with them. This innovation resulted in the establishment of three distinct paradigms, all of which have proven effective in evaluating the fish’s emotional state. The three paradigms are straightforward to implement and facilitate excellent standardization of the test. However, while it would be beneficial to explore whether these paradigms yield equivalent results, future research should aim to adopt a single paradigm for conducting cognitive bias tests. Standardizing the approach would minimize potential distortions caused by methodological differences and improve the reliability of data comparisons.

The application of the cognitive bias method has necessitated a deeper understanding of the physiology and ethology of zebrafish. Specifically, it was crucial to assess their ability to recognize colors [42,57] and verify their memory capacity before implementing the test [42,58]. This knowledge could prove valuable for future research and contribute significantly to enhancing the welfare of this species.

Despite these encouraging results, further studies are required to fully evaluate the test’s effectiveness in accurately detecting the true emotional state of zebrafish, addressing methodological bias highlighted by the SYRCLE model. The four studies that employed the five-arms device [38,39,40,41] are not devoid of methodological limitations. While these do not undermine the validity of the model, they could produce biased results.

Examining the individual studies in detail, it emerges that Buenhombre and colleagues [42] found no correlation between the results of the judgment cognitive bias test and assessments of anxiety-like behaviors. Specifically, they observed that fish subjected to a sudden gain in enrichment exhibited a negative bias compared to zebrafish kept in consistently barren conditions. Meanwhile, zebrafish exposed to a gradual loss of enrichment displayed greater anxiety-like behaviors in activity tests than those kept in constant enrichment. This inconsistency between the two tests suggests a potential limitation of the cognitive bias test, which may be more sensitive to negative changes in the affective state of zebrafish than to positive ones.

Similarly, Baciadonna and McElligott [59], in their review of research on farm animals, emphasized the difficulty of identifying rewarding stimuli compared to detecting negative ones. This challenge could be exacerbated when the area associated with the negative stimulus during training lacks punishment, potentially functioning as a neutral rather than a negative stimulus. Such nuances could confound judgment bias test results.

Individual differences—such as variations in sex, personality, or genetic strain—can also influence cognitive bias test outcomes. For example, declines in physical condition and/or lifespan, as indicated by telomerase activity, have been associated with more pessimistic judgment biases [40]. This suggests that cognitive bias tests can provide insights not only into emotional states but also into an animal’s health status, an essential factor in welfare evaluation. Additionally, stress-related impacts on reproduction can be detected using this test: pessimistic female zebrafish exhibit larger vitellogenic areas compared to their optimistic counterparts [39].

Additional limitations and points of caution in the use of cognitive bias testing align with those highlighted by Mendl et al. [6] for other species. These include variations in feeding motivation, general activity levels, and learning processes such as latent learning. Feeding motivation and general activity can be influenced by pharmacological treatments, which are often employed to assess their impact on an animal’s emotional state. Furthermore, animals tend to learn general rules about stimulus–outcome contingencies, and this latent learning can affect their responses in judgment bias tests, potentially confounding the results.

Poor reporting and insufficient use of methods to mitigate the risk of bias are quite serious concerns in the reviewed literature on cognitive bias testing. Our assessment of reporting quality and risk of bias reveals significant gaps, such as the omission of details about randomization to treatment groups, random housing, and blinding during outcome assessment. It is likely that these omissions reflect a lack of proper mitigation of these potential biases, rather than just inadequate reporting. Clearer and more consistent reporting is urgently needed throughout experiments, from sequence generation to housing randomization, and ensuring that all animals are included in the analysis.

## 6. Conclusions

All paradigms employed in the six studies cited have demonstrated their effectiveness in identifying positive or negative affective states, whether influenced by environmental factors or not. Both genetically and environmentally driven personality traits form the foundation of an individual’s affective state, which in turn underpins their welfare and quality of life. As a result, these tests hold significant potential for assessing the welfare of zebrafish.

The affective state may also influence the results of behavioral tests, highlighting the importance of evaluating personality traits before subjecting experimental animals to such protocols.

In conclusion, cognitive bias tests have proven effective for identifying personality differences influenced by genetics or environmental conditions that shape emotional states. These methods represent a valuable tool for welfare assessment in zebrafish. However, further research is necessary to evaluate the repeatability of results obtained through cognitive bias testing. Future studies should aim to eliminate methodological biases through a careful application of the SYRCLE methodology, ensuring greater reliability and consistency in findings.

## Figures and Tables

**Table 1:** A chronological list of papers was found to be consistent with the aim of the review after the selection process.

N	Title	Authors	Bibliography
1	Cognitive bias test as a tool for assessing fish welfare.	Wojtas et al., 2015	[41]
2	Insensitivity to reward shifts in zebrafish (*Danio rerio*) and implications for assessing affective states.	Tan et al., 2020	[43]
3	Short telomeres drive pessimistic judgement bias in zebrafish.	Espigares et al., 2021	[40]
4	A behavioral assay to investigate judgment bias in zebrafish.	Espigares et al., 2022a	[38]
5	Pessimistic cognitive bias is associated with enhanced reproductive investment in female zebrafish.	Espigares et al., 2022b	[39]
6	Structural environmental enrichment and the way it is offered influence cognitive judgement bias and anxiety-like behaviors in zebrafish	Buenhombre et al., 2023	[42]

**Table 2:** Risk of bias of included studies. SYRCLE risk-of-bias tool. A: Was the allocation sequence adequately generated and applied? B: Were the groups similar at baseline or were they adjusted for confounders in the analysis? C: Was the allocation adequately concealed? D: Were the animals randomly housed during the experiment? E: Were the caregivers and/or investigators blinded from knowledge of which intervention each animal received during the experiment? F: Were animals selected at random for outcome assessment? G: Was the outcome assessor blinded? H: Were incomplete outcome data adequately addressed? I: Are reports of the study free of selective outcome reporting? J: Was the study apparently free of other problems that could result in a high risk of bias? Red colored N: high risk of bias; orange colored NC: medium risk of bias; green colored Y: low risk of bias.

Authors	A	B	C	D	E	F	G	H	I	J
Wojtas et al., 2015 [41]	N	NC	N	NC	N	NC	NC	NC	N	N
Tan et al., 2020 [43]	NC	N	N	NC	N	NC	NC	Y	Y	Y
Espigares et al., 2021 [40]	NC	Y	NC	NC	N	NC	Y	Y	Y	Y
Espigares et al., 2022a [38]	N	NC	Y	NC	Y	NC	NC	NC	Y	N
Espigares et al., 2022b [39]	NC	Y	N	NC	N	Y	NC	Y	Y	Y
Buenhombre et al., 2023 [42]	NC	Y	N	NC	N	N	Y	Y	Y	N

## Data Availability

The original contributions presented in the study are included in the article; further inquiries can be directed to the corresponding author.
